# COLGALT2 is overexpressed in ovarian cancer and interacts with PLOD3

**DOI:** 10.1002/ctm2.370

**Published:** 2021-03-24

**Authors:** Ting Guo, Bin Li, Yu Kang, Chao Gu, Fang Fang, Xiuying Chen, Xiaocheng Liu, Guo Lu, Chenchen Feng, Congjian Xu

**Affiliations:** ^1^ Department of Gynecology Obstetrics and Gynecology Hospital of Fudan University Shanghai People's Republic of China; ^2^ Department of Urology, Huahsan Hospital Fudan University Shanghai People's Republic of China

To the Editor:

We here report collagen beta(1‐O) galactosyltransferase 2 (COLGALT2) is overexpressed in ovarian cancer (OvCa) and interacts with procollagen‐lysine,2‐oxoglutarate 5‐dioxygenase 3 (PLOD3).

OvCa is a major threat to women's health. We exploited The Cancer Genome Atlas dataset and identified focal gain of 1q25.3 that occurred in over 50% of OvCa cases (Figure 1A). 1q25.3 Harbored protumorigenic genes in breast cancer^1^ on which we identified COLGALT2 whose mRNA expression was higher in copy number gained cases (Figure 1B). We then performed immunohistochemistry (IHC) in a tissue microarray (TMA) containing 80 samples of primary ovarian cancer, 10 normal ovary tissues, and 10 metastatic lesions (Table S1). We found significantly higher COLGALT2 expression in both high‐grade (HG) and low‐grade (LG) serous ovarian cancer (SOC), the most common subtype of OvCa, compared with in healthy ovary tissue (Figure 2A). Whereas COLGALT2 expression in primary tumors did not differ by stage, metastasis, nodal status (Figure 1C), its expression was significantly higher in metastatic lesion (Figure 2B).

**FIGURE 1:**
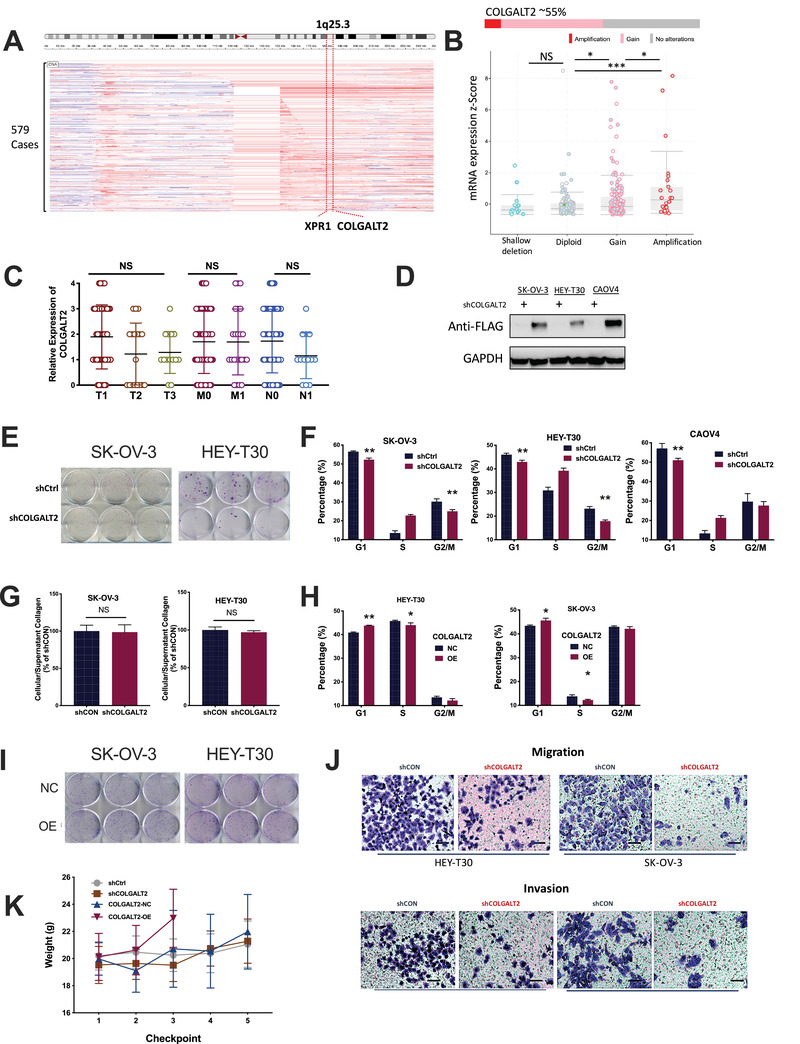
The 1q25.3 is amplified in ovarian cancer (OvCa) and targets COLGALT2 overexpression. Reproduced from TCGA fire hose legacy datasets, shown are (A) chromosomal copy number alteration at 1q25.3 from TCGA OvCa dataset; (B) relation between copy number and mRNA expression of COLGALT2 in OvCa. Immunohistochemical (IHC) staining of tissue microarray of OvCa showing (C) IHC scoring of OvCa of different stages (T), lymph node involvement (N), and metastasis (M). (D) Protein level of COLGALT2 tagged with FLAG in OvCa cell lines with shCOLGALT2 or control; (E) representative images showing colony formation of OvCa cell with shCOLGALT2 or control; flow cytometry showing (F) cell cycle profile in OvCa cell lines with COLGALT2 silencing or control; (G) pulse‐chase assay showing alteration of cellular and supernatant collagen in two OvCa cell lines with COLGALT2 silencing or control; flow cytometry showing (H) cell cycle profile and in OvCa cell lines with COLGALT2 overexpression (OE) or NC; (I) representative images showing colony formation of OvCa cell with (OE) or without (NC) overexpression of COLGALT2; (J) representative images of transwell assays of OvCa cell with shCOLGALT2 or control; xenograft mouse model using SK‐OV‐3 cells with COLGALT2 knockdown (sh) or OE and control (*N* = 10 in each group) showing (K) weight monitoring at each checkpoint (4 days) with COLGALT2‐OE group sacrificed at third checkpoint date (*p < .05; **p < .01; ***p < .001; ****p < .0001)

**FIGURE 2:**
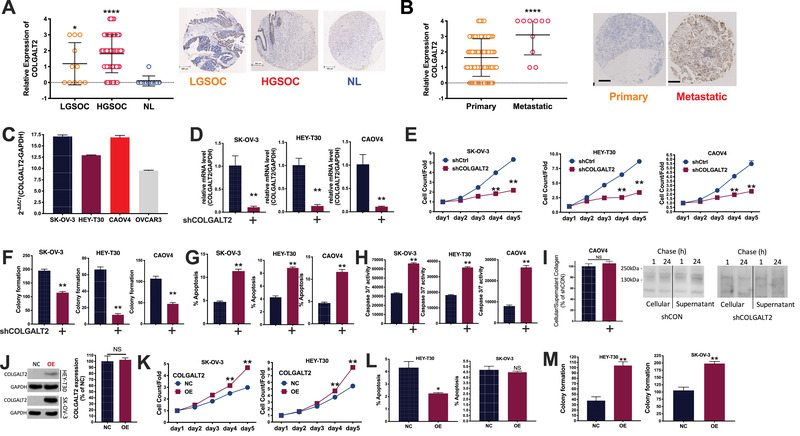
COLGALT2 is overexpressed in ovarian cancer (OvCa). (A) Immunohistochemical (IHC) staining of tissue microarray of OvCa showing IHC scoring (left panel) and representative IHC image of low‐grade and high‐grade serous ovarian cancer (LGSOC and HGSOC) and normal (NL) ovarian epithelium (right panels) (error bar = 200 μm); and (B) IHC scoring of OvCa of primary or metastatic lesion with representative IHC images (error bar = 100 μm); (C) constitutional mRNA expression of COLGALT2 in four OvCa cell lines; (D) mRNA expression of COLGALT2 in OvCa cell lines with shCOLGALT2 or control; (E) proliferation detected using Celigo in OvCa cell lines with COLGALT2 silencing or control; (F) colony formation in OvCa cell lines with COLGALT2 silencing or control; flow cytometry showing (G) cell apoptosis in OvCa cell lines with COLGALT2 silencing or control; (H) caspase activity assay showing caspase3/7 in OvCa cell lines with COLGALT2 silencing or control; (I) pulse‐chase assay showing alteration of cellular and supernatant collagen in CAOV4 cells with COLGALT2 silencing or control; (J) Western blotting showing COLGALT2 overexpression (OE) efficacy in SKOV3 and HEY‐T30 cells, and quantitative PCR showing COLGALT2 expression in CAOV4 cells; (K) proliferation detected using Celigo in OvCa cell lines with COLGALT2 OE or control (NC); flow cytometry showing (L) cell apoptosis in OvCa cell lines with COLGALT2 OE or NC; (M) colony formation in OvCa cell lines with COLGALT2 OE or NC (*p < .05; **p < .01; ***p < .001; ****p < .0001)

We next measured constitutive expression of COLGALT2 in four OvCa cell lines and the ones with higher expression (SKOV3 and HEY‐T30) and the one with both COLGALT2 amplification and overexpression (CAOV4) were chosen for further study (Figure 2C). We first identified satisfactory knockdown (KD) of the gene in all three cell lines (Figure 2D). Endogenous COLGALT2 was difficult to be captured directly on Western blotting and a FLAG tag was used (Figure 1D). COLGALT2‐KD significantly decreased proliferation (Figure 2E) and colony formation in all three cell lines (Figures 1E and 2F). COLGALT2‐KD induced significantly less cell population in G1 and G2 phases (Figure 1F). COLGALT2‐KD significantly increased cell apoptosis with significant increase of caspase 3/7 activity (Figure 2G,H). COLGALT2 harbors procollagen galactosyltransferase activity and participates in collagen chain trimerization and degradation of the extracellular matrix.^2^ We therefore used pulse‐chase assay to measure collagen level. COLGALT2‐KD did not alter intra‐ or extra‐collagen level in all three cells (Figures 1G and 2I). We then overexpressed (OE) COLGALT2 to reversely validate its role in OvCa (Figure 2J). Interestingly, COLGALT2 expression could not be increased in CAOV4 cells, possibly due to copy number gain of the gene (Figure 2J). We thus only used SKOV3 and HEY‐T30 cells in overexpression assays. COLGALT2‐OE significantly increased proliferation of both OvCa cells (Figure 2K). COLGALT2‐OE increased cell population in G1 phase and reduced population in S phase (Figure 1H). Unlike KD scenario, COLGALT2‐OE only decreased cell apoptosis in HEY‐T30 cells but not in SK‐OV‐3 cells (Figure 2L). The discrepancy in apoptosis could result from the single staining strategy (Annexin V) we used in flow cytometry (Supporting Methods) that failed to distinguish early and late apoptotic cells. Both cells showed significantly increased colony formation in the presence of COLGALT2‐OE (Figure 1I,M).

To identify interacting partners with COLGALT2, we performed proteomic assays in SK‐OV‐3 cells with COLGALT2‐OE. The mass spectrometry (MS) yielded a series of candidate proteins that showed strong network in OvCa (Figure 3A). Amongst select proteins of interest, Co‐IP assay showed that only PLOD3 was pulled down by 3 × Flag‐COLGALT2 (Figure 3B). In TCGA cohort, we found that COLGALT2 expression showed significant correlation with PLOD3 expression (Figure 3C). Similarly, protein levels of COLGALT2 and PLOD3 showed significant correlation in OvCa cell lines (Figure 3D). Of note, higher PLOD3 expression was associated with worsened prognosis in OvCa patients either with automatically designated expression cutoff or grouped by top or lower 35% of PLOD3 expression (Figure 3E). PLOD3 has been reported to mediate invasiveness of cancer cells. We performed GSEA analysis in OvCa cases with COLGALT2 copy number gain and found significant gene enrichment in cell adhesion gene set (Figure 3F). Similar result was also obtained when analyzed using NET‐GE showing cell adhesion amongst top five altered gene sets in COLGALT2 amplified cases (Figure 3G). Using transwell assays, we found significantly decreased cell migration and invasion when COLGALT2 was knocked down (Figures 1J and 3H).

**FIGURE 3:**
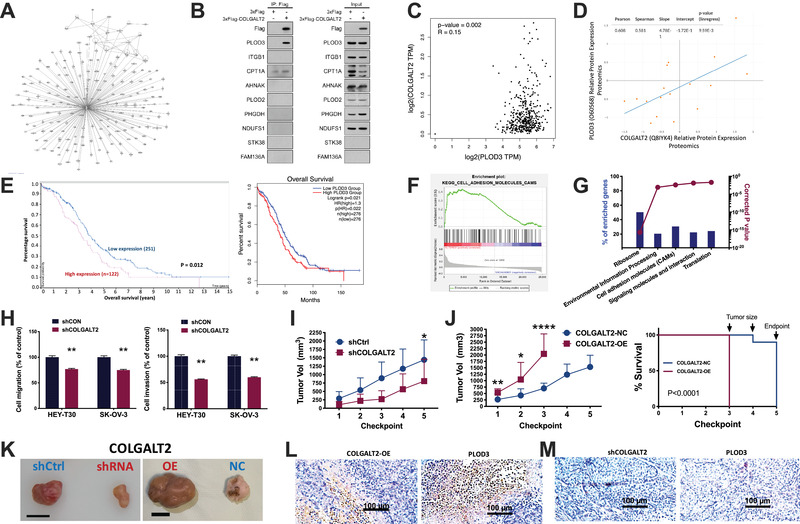
COLGALT2 interacts with PLOD3. (A) Mass spectrometry showing protein interacting with COLGALT2 in SK‐OV‐3 cells with COLGALT2‐OE, and (B) co‐immunoprecipitation of proteins of interest; (C) reproduced from TCGA fire hose legacy OvCa dataset, shown are correlation between mRNA expressions of COLGALT2 and PLOD3; (D) reproduction using DepMap showing correlation between mRNA expressions of COLGALT2 and PLOD3 in OvCa cell lines; reproduction using Human Protein Atlas of TCGA OvCa dataset shown are (E) overall survival in patients with higher or lower PLOD3 expression either by automatically designated cutoff by Human Protein Atlas, or by 35% of upper or lower PLOD3 expression, and gene expression enrichment and functional annotation using (F) GSEA and (G) NET‐GE; (H) transwell assays showing cell migration and invasion of OvCa cells with shCOLGAL2 and control; xenograft mouse model using SK‐OV‐3 cells with COLGALT2 knockdown (sh) or overexpression (OE) and control (*N* = 10 in each group) showing tumor size monitoring at each checkpoint (4 days) in (I) COLGALT2 knockdown group and in (J) COLGALT2 OE group, in which mice were sacrificed at third checkpoint date due to tumors reaching cutoff size of 2000 mm^3^, thus generating a Kaplan–Meier curve compared using log‐rank test; all xenograft tumors extracted showing (K) representative size comparison at endpoint (error bar = 1 cm) and representative immunohistochemical staining of COLGALT2 and PLOD3 in the same sample in (L) overexpression group (error bar = 100 μm) and (M) knockdown group (error bar = 100 μm) (*p < .05; **p < .01; ***p < .001; ****p < .0001)

In SK‐OV‐3 xenograft models, we found no significant toxicity in mice (Figure 1K). By the end of the fifth checkpoint date, tumor volume in COLGALT2‐KD was significantly less than that in the control group (Figure 3I). Tumors with COLGALT2‐OE however grew so fast and reached cutoff volume of 2000 mm^3^ on the third checkpoint date and mice were thus sacrificed, showing not only significantly larger tumors but also shortened survival to the study endpoint (Figure 3J,K). IHC of xenograft tumors validated corresponding COLGALT2 level in OE and KD groups, respectively (Figure 3L,M). We then semiquantitatively evaluated IHC intensity of COLGALT2 and PLOD3 in sections of human OvCa TMA and xenograft models using scoring system we developed previously.^3^ Expression of COLGALT2 showed strong positive correlation with that of PLOD3 in both TMA and in vivo tumors (Figure 3L,M; Table 1).

**TABLE 1:** Correlation between immunohistochemical scores of COLGALT2 and PLOD3 in ovarian cancer samples and xenograft tumors with SK‐OV‐3 cells

	**In tissue (*N* = 90)**		**In vivo (*N* = 40)**	
	**COLGALT2**		**COLGALT2**	
	Spearman *R*	*p*‐Value	Spearman *R*	*p*‐Value
**PLOD3**	0.9262	<.0001	0.8361	<.0001

COLGALT2 is an important paralog of COLGALT1 and promotes maturation of collagen.^2^ Our finding that COLGALT2 is higher in metastatic lesion is supported in a latest study in osteosarcoma.^4^ Unlike reported in another landmark study of COLGALTs in osteosarcoma that showed collagen accumulation in cancer cells lacking COLGALT1,^5^ we did not find alteration in collagen, neither intra‐ nor extracellular. We speculate modification of collagen by COLGALT2 was qualitative, possibly with PLOD3 as we discovered. During collagen synthesis, hydroxyproline undergoes lysyl hydroxylation with PLOD‐encoded lysyl hydroxylase (LH) to form a triple‐helix procollagen from the C‐terminal to the N‐terminal before entering extracellular matrix.^6^ The LH3 protein encoded by PLOD3 shares galactosyl hydroxylysine‐glucosyltransferase (GGT) and hydroxy lysyl galactosyltransferase (GLT) functions that facilitate the high‐level glycosylation of type IV and type VI collagen^7^ and thus promotes migration, invasion, and metastasis.^7^ We recently showed PLOD3 is correlated with advanced tumor feature of OvCa.^3^ COLGALT1 was shown to interact with PLOD3, both of which have been shown to co‐localize to endoplasmic reticulum together with mannose binding lectin.^8^ We thus postulate that COLGALT2 and PLOD3 could play similar roles in OvCa, and mechanistic analysis is undergoing.

In conclusion, we here performed comprehensive assays and showed that OLGALT2 is functionally gained in OvCa and plays protumorigenic roles. COLGALT2 interacts with PLOD3 and promotes invasiveness (Figure 4).

**FIGURE 4:**
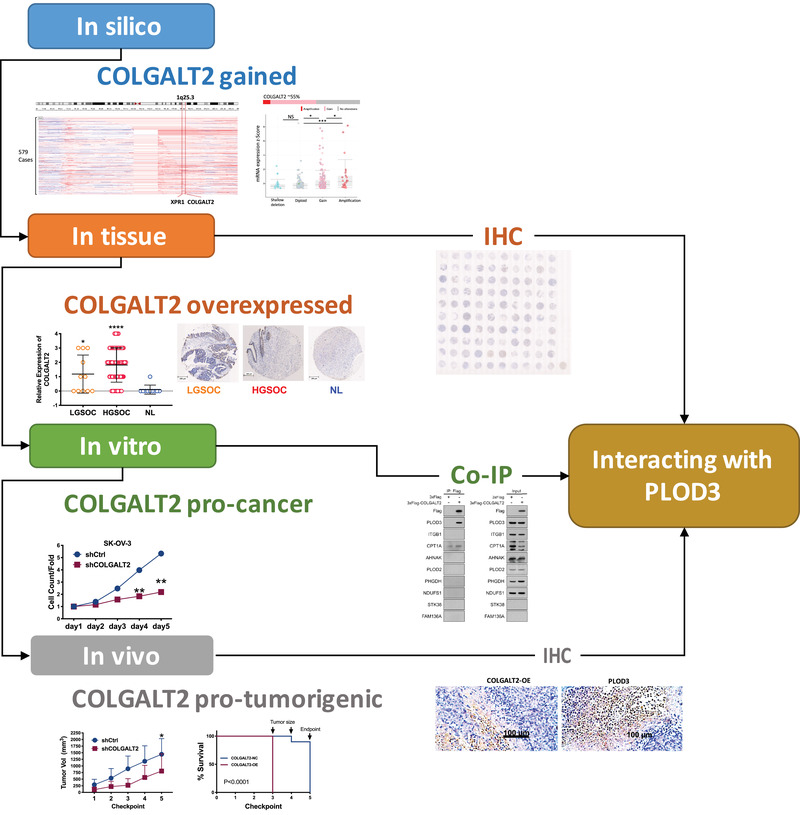
Schematic diagram of the workflow of current study

## ETHICS STATEMENT

Commercially available TMA sections were de‐identified and preconsented by the manufacturer. Rest of our study did not involve human tissue or participants. Animal ethics were approved by Genechem and Department of Laboratory Animal Science of Fudan University, where the experiments were conducted.

## CONFLICT OF INTEREST

The authors declare that there is no conflict of interest.

## FUNDING INFORMATION

National Natural Science Foundation of China, Grant/Award Number: 81602288; National Key R&D Program of China, Grant/Award Number: 2016YFC1303100; Research Project of Shanghai Municipal Health and Family Planning Commission, Grant/Award Number: 20174Y0046.

## AUTHOR CONTRIBUTIONS

Ting Guo and Chenchen Feng conceived the presented idea. Congjian Xu developed the theory and performed the computations. Bin Li, Yu Kang, Chao Gu, Fang Fang, Xiuying Chen, Xiaocheng Liu, and Guo Lu verified the analytical methods. Ting Guo, Congjian Xu, and Chenchen Feng supervised the findings of this work. All authors discussed the results and contributed to the final manuscript.

## Supporting information

Supporting Information

## References

[1] Wiechec E , Overgaard J , Kjeldsen E , Hansen LL . Chromosome 1q25.3 copy number alterations in primary breast cancers detected by multiplex ligation‐dependent probe amplification and allelic imbalance assays and its comparison with fluorescent in situ hybridization assays. Cell Oncol. 2012;36(2):113‐120. 10.1007/s13402-012-0117-1 PMC13012671 23248035

[2] Hennet T . Collagen glycosylation. Curr Opin Struct Biol. 2019;56:131‐138. 30822656 10.1016/j.sbi.2019.01.015

[3] Guo T , Gu C , Li B , Xu C . PLODs are overexpressed in ovarian cancer and are associated with gap junctions via connexin 43. Lab Invest. 2021. 10.1038/s41374-021-00533-5 33483598

[4] Wang Y , Chu Y , Li K , et al. Exosomes secreted by adipose‐derived mesenchymal stem cells foster metastasis and osteosarcoma proliferation by increasing COLGALT2 expression. Front Cell Dev Biol. 2020;8:353. 32523950 10.3389/fcell.2020.00353 PMC7262406

[5] Baumann S , Hennet T . Collagen accumulation in osteosarcoma cells lacking GLT25D1 collagen galactosyltransferase. J Biol Chem. 2016;291(35):18514‐18524. 27402836 10.1074/jbc.M116.723379 PMC5000096

[6] Shao S , Zhang X , Duan L , et al. Lysyl hydroxylase inhibition by minoxidil blocks collagen deposition and prevents pulmonary fibrosis via TGF‐beta(1)/Smad3 signaling pathway. Med Sci Monit. 2018;24:8592‐8601. 30481795 10.12659/MSM.910761 PMC6278642

[7] Qi Y , Xu R . Roles of PLODs in collagen synthesis and cancer progression. Front Cell Dev Biol. 2018;6:66. 30003082 10.3389/fcell.2018.00066 PMC6031748

[8] Liefhebber JMP , Punt S , Spaan WJM , van Leeuwen HC . The human collagen beta(1‐O)galactosyltransferase, GLT25D1, is a soluble endoplasmic reticulum localized protein. BMC Cell Biol. 2010;11:33. 20470363 10.1186/1471-2121-11-33 PMC2877668

