# Supplementary material for: COLGALT2 is overexpressed in ovarian cancer and interacts with PLOD3
Source: Clin Transl Med. 2021 Mar 24;11(3):e370. doi: 10.1002/ctm2.370 (PMC7989968; doi:10.1002/ctm2.370)
Supplement: Supplementary file 1 — Supporting Information [file CTM2-11-e370-s001.pdf]

Supplementary file

COLGALT2 is overexpressed in ovarian cancer and interacts with PLOD3

Running title: COLGALT2 promotes OvCa

Ting Guo[1], Bin Li[1], Yu Kang[1], Chao Gu[1], Fang Fang[1], Xiuying Chen[1], Xiaocheng Liu[1], Guo Lu[1], Chenchen Feng[2] and Congjian Xu[1]

[1]Department of Gynecology, Obstetrics and Gynecology Hospital of Fudan University, Shanghai, PR China
[2]Department of Urology, Huashan Hospital, Fudan University, Shanghai, PR China

Supplementary Table 1. Pathologic characteristics of the tissue microarray used in the study.

		N
**Cancer**		
**Primary**	(Adenocarcinoma)	
	Clear cell	5
	Serous papillary grade I	10
	Serous papillary grade II	12
	Serous papillary grade III	24
	Serous grade I	1
	Serous grade II	2
	Serous grade III	15
	Mucus grade I	1
	Mucus grade III	8
	Endometrioid	2
**Metastatic**	(Adenocarcinoma)	10
**Normal**	(Adjacent)	10

Supplementary Table 2. Antibody used in western blotting of Co-IP.

Antibody	Co.	Cat.	Species	Dilution	MW
Flag	Sigma	F1804	Mouse	1/1000	~90 kDa
NDUFS1	abcam	ab169540	rabbit	1/1000	75 kDa
PHGDH	proteintech	14719-1-AP	rabbit	1/1000	57 kDa
STK38	proteintech	11105-1-AP	rabbit	1/500	55 kD
CPT1A	proteintech	15184-1-AP	rabbit	1/1000	86 kDa
ITGB1	abcam	ab52971	rabbit	1/1000	140-150 (88) kDa
PLOD3	proteintech	11027-1-AP	rabbit	1/1000	80-85 kDa
PLOD2	proteintech	21214-1-AP	rabbit	1/500	85 kDa
AHNAK	proteintech	16637-1-AP	rabbit	1/500	629 kDa
FAM136A	novus	NBP1-82226	rabbit	0.4 ug/ml	16 kDa

Supplementary methods

In silico analysis

Reproduction of TCGA OvCa dataset was performed on the cBioPortal online platform (http://www.cbioportal.org/) with the selection of Firehose Legacy subsets [1, 2]. Copy number variance (CNV) dataset was used with GISTIC value of >2 designated as amplification and between 1 and 2 designated as gain. XPR1 was queried to profile focal CNV of 1q25.3 across all TCGA cancer types. COLGALT2 (Collagen Beta(1-O) Galactosyltransferase 2) was queried in OvCa dataset. Gene enrichment analysis for COLGALT2-gained cases was first analyzed using the NETwork-based Gene Enrichment (http://net-ge.biocomp.unibo.it/enrich) and then validated using the GSEA approach [3] with mRNA expression data (RNA seq V2) retrieved from TCGA. Correlations of mRNA expression in tumor tissues were also analyzed and plotted with cBioPortal. Correlations of mRNA expression in OvCa cell lines were analyzed and plotted with DepMap Portal online platform (https://depmap.org/portal/), which integrated genomic data from various high throughput sequence resources. Survival analysis for PLOD3 (Procollagen-lysine,2-oxoglutarate 5-dioxygenase 3) was performed at Human Protein Atlas platform (https://www.proteinatlas.org/). Expression cutoff for PLOD3 (high vs. low) was automatically designated by the platform in the survival analysis. The Harmonizome platform was used to examine copy number of COLGALT2 in OV cell lines (http://amp.pharm.mssm.edu/Harmonizome/).

Tissue microarray (TMA) and immunohistochemistry (IHC)

TMA sections of ovarian cancer and normal tissues were purchased from Genechem Co. (Shanghai, China). Section was de-identified and only information on cancer subtype, TNM stage, and tumor grade were available. A standard IHC protocol was followed. Briefly, section was deparaffinized after incubation for 30 min. Before graded dehydration, section was immersed in xylene for 15 min and 1:1 of xylene and alcohol for 10 min. 3% of hydrogen peroxide was used for blockade at room temperature. Antigen recovery was prepared in 0.01M sodium citrate buffer solution (pH 6.0) in a microwave for 20 min. After cooling, 10% serum in TBS was used for blockade. The primary antibody for COLGALT2 (Abcam, ab122193, at 1:100) was applied overnight. After rinsing twice with TBS, secondary antibody was applied. After rinsing 4 times, section was stained with Vulcan Fast Red Chromogen kit, then with DAB and subsequently with hematoxylin. Sections were finalized by graded dehydration and were mounted for observation. IHC scoring for each sample was the product of intensity and extensity of the immunopositive cells on membrane and cytosol. Intensity score ranged from 0 to 3 representing from no staining to brown. Extensity score was as follows: 0 for negative, 1 for 1-25% cells stained, 2 for 26%-50%, 3 for 51-75% and 4 76%-100%[4].

Cell culture and RNA interference (RNAi)

HEY-T30, SK-OV-3 and OVCAR3 ovarian cancer cells were obtained from Genechem and CAOV-4 cells were obtained from Chinese Academy of Science. Cells were cultured in RPMI-1640 medium supplemented with 10% of FBS. Target for RNAi against COLGALT2 was TAGAGAAGACTCTTGTAAT. The lentiviral vector was designed to resist puromycin. Transfection was performed using pHelper 2.0 in 293T cells followed by virus collection. Infection was conducted when cells reached 80% of confluence. After incubation for 72 h, positive clones were selected by puromycin supplement and control vectors were generated with similar approach (sequence of TTCTCCGAACGTGTCACGT). Overexpression (OE) of COLGALT2 was realized by

lentiviral delivery using polybrene system. Quantitative PCR was performed to examine the RNAi effect and inherent COLGALT2 expression level in different OvCa cell lines. Primers were as follows: forward, CGA GGG AAA AAT GGT CAG ACT AC; reverse, TGA TTC CGC ACC AGA AAT TAG AA.

Proliferation assay

A dynamic proliferation monitoring system Celigo was used. After viral infection, approximately 2500 cells/well were seeded in 96-well plate. From 24 h to 120 h, cells were examined per day. Readings were normalized to control.

Flow cytometry

The FASCanto flow cytometry system was used to measure cell cycle and apoptotic profiles. For cell cycle analysis, cells were fixed using cold ethanol and later treated with cell cycle staining buffer. For apoptosis, cells were applied with Annexin V and apoptotic cells were defined as sum of early and late apoptotic cells.

Caspase assay

Established protocol was followed as per Promega Caspase-Glo kit. Cells were seeded in 96-well plate and cultured for 3 days. Cells were resuspended at 1×10^4 cells/ well and 100 µl of pre-mixed Caspase-Glo reaction fluid was added. After gentle shaking, cells were subject to a plate reader.

Colony formation

72 h after viral infection, approximately 400-1000 cells were seeded in each well of a 6-well plate. Medium was changed every 3 days. Cells were fixed with 4% methanol on day 11 and subsequently stained by crystal violet.

Co-immunoprecipitation and proteomics

SK-OV-3 cells with stable COALGALT2-OE or control were prepared and examine for protein level by western blotting of Flag (Sigma, F1804, mouse, at 1:1000). Cells were rinsed with PBS twice and lysed pre-chilled. Cells were fragmented by ultrasound and protein concentration was determined by BCA method. Load EP tube with Flag beads and add protein lystes to a total of 1200 µl/tube. After incubation overnight at 4 °C, samples were centrifuged. Candidate genes were pre-selected by shotgun proteomics using high performance liquid chromatography combined with mass spectrometry (MS) using Q Executive for differentially translated proteins of interest. We designated unique peptide of 1 or above as credible proteins. Genes of interest were subject to western blotting in the IP assay and western blotting was performed (antibodies listed in **Suppl. Table 2**).

Pulse-chase analysis

The pulse-chase analysis was used to profile the impact of COLGALT2 on intra- and extra-cellular collagen. An established protocol was followed[5]. Briefly, cells were incubated overnight and room temperature and medium was supplemented with 50µg/ml ascorbate and 50 µg/ml catalase for another overnight culture. After one wash, cells were pulsed with 1 ml of medium containing 50 µg/ml ascorbate, 20µCi/ml L-[^{14}C(U)]-proline for 4 h. Chase started with medium change to complete medium containing 50 µg/ml ascorbate and 30 µg/ml L-proline. Collagens were digested in medium using 25 µg/ml pepsin in 1 mol HCL for 2 at 4 °C. Precipitation of collagen was finalized using 50% ethanol and resuspension was performed in Laemmli buffer. Separated collagen on SDS-PAGE gel was transferred to nitrocellulose membrane and a densitometry was used to quantify intensity of bands.

Transwell assays

Both invasion and migration were measured by Transwell assay. Cells were seeded in the upper chamber of the Transwell plate at the density of 1×10^6/ml, either coated (for invasion) and uncoated (for migration) with Matrigel. Upper chamber was

supplemented with serum-free media whilst the lower chamber was filled with complete medium. Cells that penetrated were stained with crystal violet and counted for number.

Xenograft mouse model

Subcutaneous tumor implantation mouse model was used to profile therapeutic effect in vivo. Approximately 10^7 SK-OV-3 cells with shCOLGALT2 or control were injected s.c. at axillary region of 10 female mice at 4 weeks of age per group. Tumors were calibrated every 4 days (checkpoint) and mice were euthanized on the fifth checkpoint unless tumors reached 2000mm^3 of size calibrated using the formula Length * Width^2 * 0.523. The overexpression modeling was performed using a similar method. All tumors harvested were paraffinized and processed with IHC for COLGALT2 and PLOD3 with methodology similar to TMA.

Statistical analysis

Statistical analysis for in silico studies were automatically performed with the platforms used, as aforementioned. Statistical analysis for in vitro assays and in vivo experiments were performed using the Prism Graphpad 7.0 for Mac. All assays were performed in triplicates with a sample size of 6. Comparisons between two groups were studied using the Mann-Whitney test for non-parametric variants (IHC scores) and using the Student's t test for parametric variants (RNA-seq reads) and using the Mann-Whitney test for non-parametric variances. The survival data was presented using the Kaplan-Meier curve and compared using the Log-rank test. The P value of < .05 was accepted as significant.

References

1. Gao J, Aksoy BA, Dogrusoz U, Dresdner G, Gross B, Sumer SO, Sun Y, Jacobsen A, Sinha R, Larsson E *et al*: **Integrative Analysis of Complex Cancer Genomics and Clinical Profiles Using the cBioPortal**. *Science Signaling* 2013, **6**(269):pl1-pl1.
2. Cerami E, Gao J, Dogrusoz U, Gross BE, Sumer SO, Aksoy BA, Jacobsen A, Byrne CJ, Heuer ML, Larsson E *et al*: **The cBio Cancer Genomics Portal: An Open Platform for Exploring Multidimensional Cancer Genomics Data: Figure 1**. *Cancer Discovery* 2012, **2**(5):401-404.
3. Subramanian A, Tamayo P, Mootha VK, Mukherjee S, Ebert BL, Gillette MA, Paulovich A, Pomeroy SL, Golub TR, Lander ES *et al*: **Gene set enrichment analysis: a knowledge-based approach for interpreting genome-wide expression profiles**. *Proc Natl Acad Sci U S A* 2005, **102**(43):15545-15550.
4. Guo T, Gu C, Li B, Xu C: **PLODs are overexpressed in ovarian cancer and are associated with gap junctions via connexin 43**. *Laboratory Investigation* 2021.
5. Baumann S, Hennet T: **Collagen Accumulation in Osteosarcoma Cells lacking GLT25D1 Collagen Galactosyltransferase**. *Journal of Biological Chemistry* 2016, **291**(35):18514-18524.
